# The Relationship of Lifestyle Risk Factors and Depression in Korean Adults: A Moderating Effect of Overall Nutritional Adequacy

**DOI:** 10.3390/nu13082626

**Published:** 2021-07-29

**Authors:** Minjeong Kang, Mingyu Joo, Haeryun Hong, Hyunsik Kang

**Affiliations:** College of Sport Science, Sungkyunkwan University, Suwon 16419, Korea; kangmin125@skku.edu (M.K.); jmg0403@naver.com (M.J.); hhr8028@skku.edu (H.H.)

**Keywords:** lifestyle risk factors, nutritional adequacy, depression, mental health, Korean adults

## Abstract

Background: Little is known regarding the role of nutrition in determining the associations between lifestyle risk factors and depression. Objectives: This study examined whether or not nutritional adequacy modulates the relationship between depression and lifestyle risk factors in Korean adults aged 18-65 years (*n* = 7446). Methods: Data were obtained from the 2016 and 2018 Korea National Health and Examination Survey. Depression, smoking, at-risk alcohol consumption, physical inactivity, sleep deprivation, and mean adequacy ratio (MAR) were assessed. Results: Individuals with two (OR = 1.960, *p* < 0.001), three (OR = 4.237, *p* < 0.001), or four (OR = 5.312, *p* < 0.001) risk factors had a significantly higher risk of depression compared to individuals with one or zero risk factor. In contrast, individuals with moderate MAR (OR = 0.607, *p* < 0.001) and high (OR = 0.698, *p* < 0.001) MAR had a lower depression risk compared to individuals with low MAR. Moderation analysis showed a moderating effect of MAR (coefficient = −0.220, *p* = 0.007) on the relationship between risk factors and depression. Conclusions: The current findings suggest that overall nutritional adequacy plays a modulating role in determining the relationship between depression and lifestyle risk factors in Korean adults.

## 1. Introduction

Depression is a common mental disorder and a leading cause of disability worldwide [1]. In South Korea, the prevalence of depression is relatively low but steadily on the rise [2]. Furthermore, among Organization for Economic Co-operation and Development countries, South Korea has the highest suicide rate at 23 deaths per 100,000 persons [3]. The estimated total economic burden of depression is rising steadily, from 4049 million USD in 2005 [4] to 1.331 billion USD in 2012 [5]. Depression in South Korea has been blamed for rising disability and surging economic burden in conjunction with the increasing rate of suicide.

There is a number of well-established risk factors associated with depression and depressive symptoms [6]. The risk factors include current or past smoking [7], heavy alcohol consumption [8], low income [9], unemployment [10], low social support [11], perceived stress [12], physical inactivity [13], sleep deprivation [14], and unhealthy diet [15]. Associations between depression and these risk factors have been reported in Korean populations [16]. Parameters of health behaviors and perceived health status are additional predictors of depression in Korean adults [17].

Nutrition is another important factor to be considered in determining the onset of depression and the severity of depressive symptoms associated with lifestyle risk factors [18]. For example, depression is associated with several nutritional parameters, including dietary intake of nutrients [19], quality of diet [20], dietary habits [21], and food security [22]. The relationship between depression and nutritional parameters has been reviewed and well summarized in previous studies [23]. Overall, the findings from previous studies suggest that nutritional adequacy, which is defined as the sufficient intake of nutrients of interest for optimal health, may play a role in the onset and maintenance of depression as well as in the severity of depressive symptoms associated with lifestyle risk factors. Therefore, examining how nutrition influences the relationship between depression and lifestyle risk factors will provide some critical information for preventive and therapeutic strategies against the mental illness. 

To the best of our knowledge, no previous research has examined the potential role of nutrition in determining the relationship between depression and lifestyle risk factors in Korean adults. The current study was conducted to investigate whether or not overall nutritional adequacy moderates the association between depression and unhealthy lifestyle factors in a representative sample of Korean adults.

## 2. Materials and Methods

### 2.1. Data Source and Study Participants

**Figure 1:**
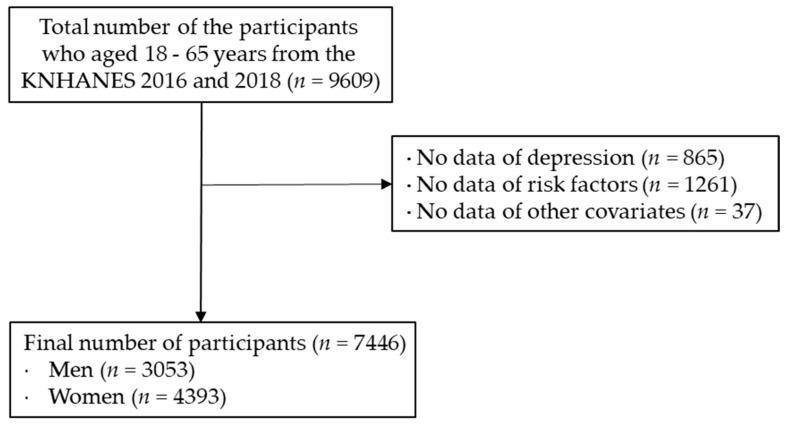
Flow chart of selection for study participants.

As illustrated in Figure 1, we initially selected respondents aged 18–65 years (*n* = 9609) from the seventh Korea National Health and Examination Survey (KNHANES) in 2016 and 2018, a nationwide survey designed to assess health and nutritional status in the Korean population. Exclusion criteria were missing information or refusal to provide information on depression scores (*n* = 1178), missing information or refusal to provide information on lifestyle risk factors (*n* = 1285), and missing information or refusal to provide information on other variables (*n* = 84). Consequently, the remaining 7446 participants (women, *n* = 4393; 59%) were included in final data analyses. A detailed description of the KNHANES study design is available elsewhere [24]. The Institutional Review Board of Korea Centers for Disease Control and Prevention reviewed and approved the KNHANES VII surveys (2018-01-03-P-A) in accordance with the Declaration of Helsinki. Informed consent was obtained from all the participants. 

### 2.2. Variables

#### 2.2.1. Patient Health Questionnaire-9

The Korean version of the Patient Health Questionnaire (PHQ)-9, which is a self-reported version of the PRIME-MD diagnostic instrument for major depressive disorders [25], was downloaded from the PHQ website (https://www.phqscreeners.com/, accessed on 10 January 2021) and used to score each of the nine items corresponding to the Diagnostic and Statistical Manual of Mental Disorders criteria as “0” (not at all) to “3” (nearly every day). The threshold for identifying major depressive disorders was 5, and its validity was previously tested and reported [26]. 

#### 2.2.2. Lifestyle Risk Factors

Lifestyle risk factors included in the study were past/current smoking, at-risk alcohol consumption, physical inactivity, and sleep deprivation. For smoking, respondents were dichotomously classified as never smokers and past or current smokers. At-risk alcohol intake was defined as having seven glasses or more (five or more for women) of alcohol per occasion two or more times per week [27]. Physical inactivity was defined as not participating in at least 150 min of moderate physical activity (PA) per week or 75 min of vigorous PA or a combination of moderate and vigorous PA (https://www.who.int/news-room/fact-sheets/detail/physical-activity, accessed on 20 March 2021). Sleep deprivation was defined as inadequate duration of sleep (<7 h) or use of hypnotic medications [28]. 

#### 2.2.3. Dietary Data

Dietary intake of macronutrients (i.e., carbohydrates, fats, and proteins) and micronutrients (i.e., vitamins A and C, thiamine, riboflavin, niacin, phosphorous, calcium, and iron) was assessed with a 24-h (h) recall method. Trained interviewers conducted computer-assisted personal interviews to assess all food items ingested during the previous 24 h. Mean adequacy ratio (MAR), which is a measure that is used to evaluate an individual’s intake of a nutrients and represents a population’s overall nutritional adequacy, was calculated using the following equation: MAR = (sum of nutrient adequacy ratio (NAR)/number of nutrients) × 100. Nutrient adequacy ratio (NAR) represents an individual’s intake of a nutrient as a percentage of the age- and sex-specific recommended dietary allowance (RDA) for that nutrient [29]. Mean adequacy ratio (MAR) and quantifies the overall nutritional adequacy of a population based on the current recommended allowance of a group of nutrients of interest. 

#### 2.2.4. Covariates

Age (continuous), sex (categorical: male or female), body mass index (quantitative), education (categorical: elementary school or lower, middle or high school, university or higher), income (quantitative), and marital status (categorical: yes or no) were included as covariates. Body mass index (BMI) was calculated as weight divided by height squared (kg/m^2^).

### 2.3. Statistics

Normality of data distribution was confirmed with QQ plotting, and the absence of multi-collinearity was assessed by variance of inflation factor (VIF). Descriptive statistics were performed with Student’s t-test and Chi-square test for continuous and categorical variables, respectively, which are presented as the mean ± standard deviation (SD) and number (n) or percentage (%), respectively. Linear regression was used to assess the relationship between PHQ-9 scores and other variables. Multivariate logistic regression was performed to estimate the odds ratios (ORs) and 95% confidence intervals (CIs) of depression by number of lifestyle risk factors and MAR level (from low to high) before and after adjustments for the covariates. To find out whether or not the strength of the relationship between lifestyle risk factors and depression risk varies according to overall nutritional adequacy, a moderation analysis with the interaction term between the risk factors and MAR (moderator) was performed with the Hayes PROCESS macro. The significance of the moderator was tested by the change of proportion concerning the explained variance (∆R2). Statistical significance was defined as *p* < 0.05. All statistical analyses were conducted using PASW SPSS WIN 27.0 (SPSS Inc., Chicago, IL, USA).

## 3. Results

Table 1 describes the characteristics of study participants by sex. Men had higher BMI, better education levels, and lower rates of marriage in comparison to women, with no differences in mean age and household income. With respect to lifestyle risk factors, men had higher rates of smoking, at-risk alcohol consumption, and sleep deprivation in conjunction with a lower rate of physical inactivity in comparison to women. In addition, men had higher intakes of carbohydrates, proteins, vitamin A, thiamine, riboflavin, niacin, phosphorous, calcium, and iron in conjunction with higher caloric intake in comparison to women. Finally, men had a lower mean PHQ-9 score in comparison to women.

Table 2 presents the outcomes of linear regression analysis for PQH-9 scores. Depression risk was positively associated with age, female, unmarried, smoking, at-risk alcohol consumption, physical inactivity, and sleep deprivation, while it was negatively associated with education level, income, and MAR.

Table 3 presents the outcomes of logistic regression for depression risk by lifestyle risk factors and MAR, respectively. Individuals with two (OR = 1.65, 95% CI = 1.24~2.20, *p* < 0.001), three (OR = 2.97, 95% CI = 2.21~3.98, *p* < 0.001), and four lifestyle risk factors (OR = 3.19, 95% CI = 2.09~4.868, *p* < 0.001) had higher risks of depression in comparison to individuals with one or zero risk factors (OR = 1). Increased ORs for the two-risk factors (*p* < 0.001), the three-risk factors (*p* < 0.001), and the four-risk factors (*p* < 0.001) remained statistically significant even after adjustments for age, sex, income, education, marital status, and MAR. On the other hand, individuals with middle 50 percentile MAR (OR = 0.45, 95% CI = 0.36~0.58, *p* < 0.001) or highest 25 percentile MAR (OR = 0.46, 95% CI = 0.34~0.62, *p* < 0.001) had significantly lower risks of depression in comparison to individuals with lowest 25 percentile MAR (OR = 1). Decreased ORs for the middle 50 percentile MAR and highest 25 percentile MAR remained statistically significant even after adjustments for age, sex, income, education, marital status, and lifestyle risk factors (i.e., smoking, at-risk alcohol consumption, physical inactivity, and sleep deprivation).

**Figure 2:**
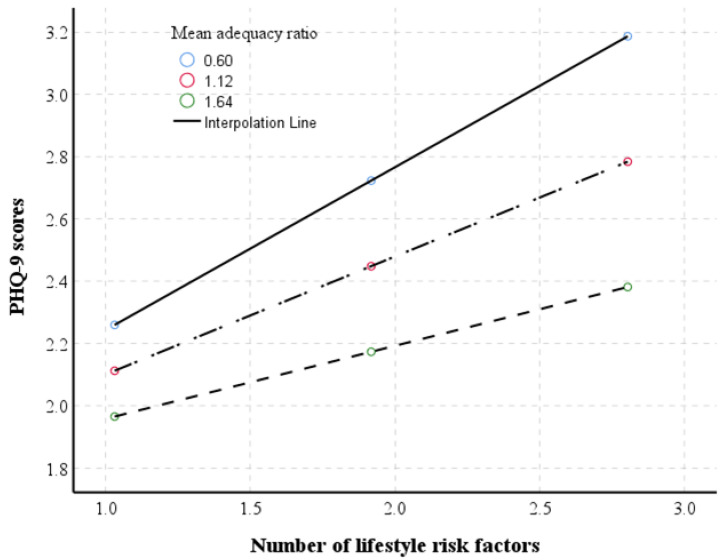
Illustration of the moderating effect of mean adequacy ratio on the relationship between lifestyle risk factors and patient health questionnaire (PHQ)-9 scores.

Table 4 presents the outcomes of a moderating analysis of MAR for the relationships between depression and lifestyle risk factors. There was a significant interaction between lifestlye risk factors and nutrition (coefficient = −0.259, 95% CI = −0.423~−0.094, *p* = 0.002) on MAR, observed by increasing a part of the explained variability up to 2% (∆R^2^ = 0.018). The moderating effect of MAR remained significant (coefficient = −0.220, 95% CI = −0.380~−0.061, *p* = 0.007) even after adjustments for the covariates, observed by further increasing a part of the explained variability up to 8% (∆R^2^ = 0.001). As illustrated in Figure 2, overall nutritional adequacy attenuates the strength of the relationship between PHQ-9 score and lifestyle risk factors—the higher was the MAR, the less steep was the slope of the relationship.

## 4. Discussion

This study examines whether or not overall nutritional adequacy modulates the relationships of depression with lifestyle risk factors (i.e., past/current smoking, at-risk alcohol consumption, physical inactivity, and sleep deprivation) in 7446 Korean adults who participated in the 2016 and 2018 KNHANES. The findings of the current study show that depression risk is positively associated with all the four risk factors and negatively associated with overall nutritional adequacy. Novel to our study is that overall nutritional adequacy attenuates the adverse effect of lifestyle risk factors on depression in Korean adults. 

Consistent with the current findings, previous studies have reported a significant association between depression and lifestyle factors in Western populations [30,31,32]. The associations between lifestyle risk factors and depression are also observed in Korean populations. In a representative sample of 4093 Korean adults who participated in the 2014 KNHANES, Jung et al. [33] showed that both hypertension and dyslipidemia were independent determinants of depression in women, and that dyslipidemia alone was an independent determinant of depression in men. Kim and Kim [34] showed that depression risk was significantly associated with metabolic syndrome risk factors in Korean adults by analyzing data (*n* = 10,459) from the 2014 and 2016 KNHANES. By analyzing data from the 2012 Korean Community Health Survey in adults aged 19 years and older (*n* = 10,081), Won et al. [35] showed that depression was significantly associated with subjective health, drinking of alcohol, smoking, hypertension, diabetes mellitus, total hours of sleep, activity impairments, and stress in Korean adults. Taken together, these previous findings support the current findings in which lifestyle risk factors are significantly associated with increased risk of depression. 

In particular, the current findings suggest the potential role of dietary intake of nutrients in determining the relationship between lifestyle risk factors and depression. By conducting a community-based study involving three age-based Australian groups, for example, Jacka et al. [36] showed that unhealthy dietary patterns were significantly and independently associated with increased depressive symptoms, especially in the oldest cohort. In the Japan Multi-Institutional Collaborative Cohort Study involving 4701 Japanese adults aged 35–69 years, Choda et al. [37] showed that depression risk was inversely associated with dietary intake of vegetables, protein, calcium (Ca), vitamin D, carotene, and n-3 high-polyunsaturated fatty acids and positively associated with monounsaturated fatty acids. In a cohort study of 13,983 Spanish university graduates, Sánchez-Villegas et al. [38] found that inadequate intake of ≥four micronutrients out of a collection of vitamins, including vitamins B1, B2, B3, B6, B12, C, A, D, E, folic acid (FA), zinc (Zn), iodine (I), selenium (Se), iron (Fe), Ca, potassium (K), phosphorus (P), magnesium (Mg), and chrome (Cr), was associated with increased risk of depression. By comparing depressive patients with their healthy counterparts, Kaner et al. [39] found that depression was significantly associated with lower intakes of micronutrients, such as vitamin A, thiamine, riboflavin, vitamin B6, folate, vitamin C, Na, K, Mg, Ca, P, Fe, Zn, and fiber. Consequently, the findings from the current and previous studies suggest that the sufficient intake of those essential nutrients may function to minimize the adverse effects of lifestyle risk factors on depression. In addition, the current findings also suggest that dietary interventions may be a promising intervention for reducing depression and/or depressive symptoms associated with lifestyle risk factors. 

Protective effects of healthy dietary intake and habits against depression risk are also observed in Korean populations. For example, Park et al. [40] reported that low dietary intake of fiber and vitamin C was significantly associated with depression risk in Korean women by analyzing data from the 2014 KNHANES (*n* = 5897). By analyzing data from the sixth (2014) and seventh (2016, 2018) KNHANES (*n* = 19,106), Yun et al. [41] found that adverse dietary intake (i.e., dietary intake of sugar, carbohydrates, sodium, and others) and habits (i.e., dining out and dining frequency) were significantly associated with depression risk in Korean adults, with age- and sex-based differences in the strength of the association. By analyzing data (*n* = 5103) from the 2018 KNHANES, Lee and Kim [42] showed that dietary intake of phosphorus, potassium, and vitamin A was inversely associated with depression risk in men, while dietary intake of phosphorus was inversely associated with depression risk in women. That study also found that eating behaviors, such as skipping lunch, frequency of daily meals, and frequency of weekly lunches out, were significantly associated with a higher prevalence of depression in Korean women only. Protective effects of dietary intake against depression were observed for various micronutrients such as riboflavin, thiamin, vitamin C, polyunsaturated fatty acids, fibers, fruits, and vegetables in Korean populations [43,44]. Taken together, findings from the current and previous studies suggest that unhealthy dietary intake and habits are significantly associated with the onset of depression and the severity of depressive symptoms in Korean adults. 

Some explanations can be given to the modulating effects of overall nutritional adequacy on the relationship between the four lifestyle risk factors and depression. First, overall nutritional adequacy may have protective effects against depression by securing sufficient dietary intake of anti-depressant and anti-oxidant nutrients [45]. Second, overall nutritional adequacy may reflect the sufficient intake of other essential nutrients needed to fulfill nutritional requirements for good mental health [46]. Third, it is also possible that overall nutritional adequacy has an indirectly protective effect against depression by minimizing clinical consequences of unhealthy behaviors such as obesity and metabolic diseases [47].

This study has some limitations. First, the cross-sectional nature of the study does not allow any cause-and-effect explanation regarding the modulating effect of overall nutritional adequacy on the relationship between depression and lifestyle risk factors. Second, a bidirectional relationship between depression and lifestyle risk factors seems possible-depression can lead to an increase in lifestyle risk factors, or vice versa, and this remains to be further addressed. Third, it is possible that the modulating effect of overall nutritional adequacy on the relationship between lifestyle risk factors and depression differ by some confounders such as age and sex. This should be addressed by including demographics as additional modulators. Fourth, the PHQ-9 used in the current study is a self-reported diagnosis of depression. Therefore, the possibility of type 2 errors or false negatives cannot be completely ruled out. Lastly, we did not consider other potential risk factors involved in depression, including brain neurotransmitters, genetic vulnerability, medications, substance misuse, and others. Caution is necessary in interpreting the relationship of depression with nutritional adequacy and lifestyle risk factors observed in the current study.

Despite limitations, this study also has strengths. First, it is a population-based study with a relatively high response rate and large sample size. “Second, to the best of our knowledge, we are the first to report the potential of overall nutritional adequacy as a modulator in determining the relationship between lifestyle risk factors and depression. At the same time, we also recognize that the interaction between lifestyle risk factors and overall nutritional adequacy on depression cannot be explained in a cause-and-effect manner due to the cross-sectional nature of this study. Consequently, a randomized controlled trial is warranted to investigate the protective role of healthy dietary intake and habits against the adverse effects of lifestyle risk factors on the onset of depression and/or the severity of depressive symptoms. Third, the relationship between lifestyle risk factors and depression may imply adverse effects secondary to the risk factors such as metabolic complications. 

## 5. Conclusions

This population-based study finds that overall nutritional adequacy can attenuate the adverse effect of lifestyle risk factors on depression in Korean adults, implying the importance of nutrition as a therapeutic strategy against the mental illness.

## Figures and Tables

**Table 1:** Characteristics of study participants.

Variable				Total (*n* = 7446)	Men (*n* = 3053)	Women (*n* = 4393)	*p* Value
Age (years)				44.3 ± 12.3	44.1 ± 12.5	44.4 ± 12.1	0.433
Body mass index (kg/m^2^)				23.9 ± 3.7	24.8 ± 3.5	23.2 ± 3.6	<0.001
Socioeconomic status							
		Income (10,000 won/month)		497.0 ± 313.2	502.9 ± 313.6	492.9 ± 312.9	0.177
		Education, n (%)					<0.001
			Lower than elementary	444 (6.0)	131 (4.4)	313 (7.2)	
			Middle/high	2785 (37.9)	996 (33.2)	1789 (41.1)	
			Over than College	4119 (56.1)	1873 (62.4)	2246 (51.7)	
		Marital status, *n* (%)					<0.001
			Unmarried	1490 (20.3)	782 (26.1)	708 (16.3)	
			Married	5858 (79.7)	2218 (73.9)	3640 (83.7)	
Lifestyle risk factors							
		Past/current smoking, *n* (%)		2820 (38.4)	2263 (75.4)	557 (12.8)	<0.001
		Heavy alcohol intake, *n* (%)		2002 (27.2)	1193 (39.8)	809 (18.6)	<0.001
		Physical inactivity, *n* (%)		6058 (82.4)	2322 (77.4)	3736 (85.9)	<0.001
		Insomnia, *n* (%)		2880 (39.2)	1225 (40.8)	1655 (38.1)	0.017
Dietary intakes							
	Caloric intake (kcal/day)			2034 ± 913	2469 ± 1001	1732 ± 704	<0.001
	CHO intake (g/day)			295.1 ± 123.7	340.1 ± 130.7	264.1 ± 108.2	<0.001
	Fat intake (g/day)			49.0 ± 35.9	59.1 ± 41.8	42.0 ± 29.3	<0.001
	Protein (g/day)			73.8 ± 41.0	89.8 ± 47.6	62.8 ± 31.3	<0.001
	Vitamin A (μgRAE/day)			407.2 ± 533.2	451.3 ± 538.2	376.9 ± 527.6	<0.001
	Vitamin C (mg/day)			84.3 ± 100.2	82.8 ± 100.4	85.3 ± 100.0	0.306
	Thiamine (mg/day)			1.7 ± 1.0	2.0 ± 1.1	1.7 ± 1.0	<0.001
	Riboflavin (mg/day)			1.6 ± 0.9	1.8 ± 1.0	1.4 ± 0.7	<0.001
	Niacin (mg/day)			15.3 ± 8.9	18.1 ± 10.2	13.4 ± 7.3	<0.001
	Phosphorus (mg/day)			1087.2 ± 506.6	1273.4 ± 563.6	958.7 ± 417.4	<0.001
	Calcium (mg/day)			507.6 ± 277.9	575.0 ± 330.2	461.1 ± 277.9	<0.001
	Iron (mg/day)			14.6 ± 9.9	16.9 ± 11.2	13.1 ± 8.6	<0.001
PHQ-9 score				2.4 ± 3.4	1.9 ± 3.0	2.8 ± 3.6	<0.001

PHQ: Patient health questionnaire.

**Table 2:** Linear regression analysis for the determinants of depression in study participants.

Variables	Beta	95% CI	*p* Value	VIF
Age	−0.023	−0.032~−0.014	<0.001	2.158
Sex	1.613	1.408~1.817	<0.001	1.819
BMI	−0.004	−0.025~0.017	0.723	1.079
Education	−0.328	−0.478~−0.178	<0.001	1.496
Income	−0.001	−0.002~−0.001	<0.001	1.100
Marital status	0.591	0.352~0.830	<0.001	1.725
Smoking	1.196	0.990~1.402	<0.001	1.746
At-risk alcohol intake	0.333	0.152~0.514	<0.001	1.172
Physical Inactivity	0.306	0.106~0.507	0.003	1.048
Inadequate sleep	0.426	0.273~0.580	<0.001	1.010
MAR	−0.226	−0.376~−0.077	0.003	1.068

CI: confidence interval; VIF: variance inflation factor; BMI: body mass index; MAR: mean adequacy ratio.

**Table 3:** Odds ratios (ORs) and 95% confidence intervals (CIs) of depression by lifestyle risk factors and nutritional adequacy ratio.

Predictors		Model 1		Model 2	
		OR (95% CI)	*p* Value	OR (95% CI)	*p* Value
Number of lifestyle risk factors					
	<1	1 (referent)		1 (referent)	
	2	1.651 (1.243~2.195)	0.001	1.960 (1.423~2.537)	<0.001
	3	2.966 (2.212~3.978)	<0.001	4.237 (3.085~5.821)	<0.001
	4	3.188 (2.088~4.868)	<0.001	5.312 (3.358~8.403)	<0.001
Mean adequacy ratio (MAR)					
	Low	1 (referent)		1 (referent)	
	Middle	0.452 (0.355~0.576)	<0.001	0.607 (0.472~0.781)	<0.001
	High	0.463 (0.344~0.621)	<0.001	0.698 (0.511~0.952)	0.023

Model 1: unadjusted; Model 2: adjusted for age, body mass index, sex, income, education, marital status, and MAR for ORs of lifestyle risk factors or adjusted for age, sex, income, education, marital status, and 4 lifestyle risk factors for ORs of MAR.

**Table 4:** Moderation analyses of mean adequacy ratio for the relationship of depression with lifestyle risk factors.

Predictors		Coefficients	SE	t	*p*	95% CI	
						Lower	Upper
Model 1 (R^2^ = 0.018, F = 44.088, *p* < 0.001)							
	LRF	0.666	0.104	6.392	<0.001	0.462	0.871
	MAR	−0.065	0.185	−0.353	0.724	−0.427	0.297
	Interaction	−0.259	0.084	−3.081	0.002	−0.423	−0.094
	R^2^ change due to the moderator = 0.001 (F = 9.494, *p* = 0.002)						
Model 2 (R^2^ = 0.077, F = 68.418, *p* < 0.001)							
	LRF	0.855	0.103	8.302	<0.001	0.653	1.057
	MAR	0.215	0.180	1.194	0.233	−0.138	0.568
	Interaction	−0.220	0.081	−2.705	0.007	−0.380	−0.061
	R^2^ change due to the moderator = 0.001 (F = 494, *p* = 0.002)						

LRF: lifestyle risk factors; MAR: mean adequacy ratio. Model 1: unadjusted; Model 2: adjusted for age, body mass index, sex, income, education, and marital status.

## Data Availability

Data can be accessible upon request to corresponding author (hkang@skku.edu).
